# Stochastic changes in gene expression promote chaotic dysregulation of homeostasis in clonal breast tumors

**DOI:** 10.1038/s42003-019-0460-0

**Published:** 2019-06-14

**Authors:** Sara J. Felts, Xiaojia Tang, Benjamin Willett, Virginia P. Van Keulen, Michael J. Hansen, Krishna R. Kalari, Larry R. Pease

**Affiliations:** 1 Department of Immunology, Mayo Clinic, Rochester, MN 55905 USA; 2 Division of Biomedical Statistics and Informatics, Mayo Clinic, Rochester, MN 55905 USA

**Keywords:** Breast cancer, Cancer epigenetics, Cancer models

## Abstract

Cells within tumors vary in phenotype as a result of changes in gene expression caused by a variety of mechanisms, permitting cancers to evolve under selective pressures from immune and other homeostatic processes. Earlier, we traced apparent losses in heterozygosity (LOH) of spontaneous breast tumors from first generation (F1) intercrossed mice to atypical epigenetic modifications in the structure of DNA across the tumor genomes. Here, we describe a parallel pattern of LOH in gene expression, revealed through quantitation of parental alleles across a population of clonal tumors. We found variegated patterns of LOH, based on allelic ratio outliers in hundreds of genes, enriched in regulatory pathways typically co-opted by tumors. The frequency of outliers was correlated with transcriptional repression of a large set of homozygous genes. These findings suggest stochastic losses in gene expression across the genome of tumors generate phenotypic variation among cells, allowing clonal selection during tumor development.

## Abstract (web summary)

Sara Felts et al. present atypical losses of heterozygosity in genes expressed in breast tumors of first-generation intercrossed mice. They find evidence suggesting sporadic epigenetic errors causing dysregulated cellular homeostasis in tumors.

## Introduction

Key features of tumorigenesis include an accumulation of chromosomal, genetic, and epigenetic aberrations that result in losses of cellular control mechanisms and cancerous tissues with evolving phenotypes. Cancer genome studies provide robust data sets for understanding mutation burden important for patient stratification, the identification of targetable intervention strategies, and lending insight into cancer evolution^1,2^. However, as more minor genomic variants are cataloged with small associations with tumor development and disease outcomes^3–7^, there is a need to understand how these minor changes contribute in concert to cancer phenotypes. Our recent studies indicate that small differences in gene expression integrated across many genes can specify cellular phenotypes^8^. How tumor phenotypes are shaped by such integrative events needs further investigation. In vivo models of cancer often employ spontaneous or induced tumor formation in inbred mice. As humans are an outbred population characterized by genome-wide heterozygosity, important aspects of the evolution and behavior of human cancers can be modeled only using heterozygous animals.

Germline single nucleotide variants (SNVs) differentiating strains of laboratory mice can be used to track genetic changes at the level of genomic DNA, as well as at the level of tissue-specific transcriptomes^9–12^. In a previous study using an F1 model of spontaneous breast cancer, we found evidence of previously unappreciated widespread epigenetic modifications in tumor DNA marked by polymorphisms^13^. Tumorigenesis is initiated by oncogenic *HER2* (rat *neu*) and ensues rapidly in this model^14^. As a test of whether stochastic epigenetic events had an impact on tumor gene expression, we used RNAseq to quantitate the balance (or imbalance) of germline-encoded, expressed single nucleotide variants (eSNVs) in tumor mRNA. Eighteen of twenty characterized tumors were dominated by homozygous X-chromosome inactivation, providing a strong indication of their clonal evolution. Within these tumor clones, we found unique, statistically significant variegated patterns of LOH (V-LOH) representing stochastic over- or under-representation of eSNVs. As these events were manifest in alleles integral to multiple regulatory pathways and each tumor had many such events, the data illustrate how various combinations of small quantitative changes can be selected to drive clonal evolution of tumors in the context of a driver mutation. Furthermore, a strong negative correlation between the frequency of outliers in the ratio of expressed alleles and the levels of expression for hundreds of genes in these tumors indicates that the underlying mechanism operates to decrease expression of genes whose normal functions are to maintain tissue homeostasis.

## Results

We used RNAseq to quantify the relative contribution of germline parental alleles to genes expressed in [FVB/J x BALB/c-(neuT)] F1 spontaneous breast tumor samples. In this model, all oncogene-driven tumors^14–16^ share a common germline genetic origin, yet develop into genotypically diverse tumors over the course of 3–4 months^13^. Gene expression data obtained by RNAseq analysis of 20 independent late-stage tumors were aligned to the mouse reference genome (mm10, C57BL/6); FVB and BALB allele counts were measured separately for over 56,300 potential variants. Approximately 12,400 eSNVs met a threshold of 15 raw read counts and were considered further. All allelic ratios were converted to BALB/c-allele frequency (CAF) for ease of discussion and were visualized along each mouse chromosome (Chr1-Chr19 plus ChrX) as mean CAFs for individual eSNVs (Supplementary Fig. 1). A 1:1 ratio of expressed parental alleles was typical, with occasional locus-associated imbalances across all tumors, many of which reflect known imprinting or demonstrable mapping artifacts. We first focused on genes where the predominant phenotype among all tumors deviated substantially from the expected 1:1 ratio of expressed alleles.

**Fig. 1:**
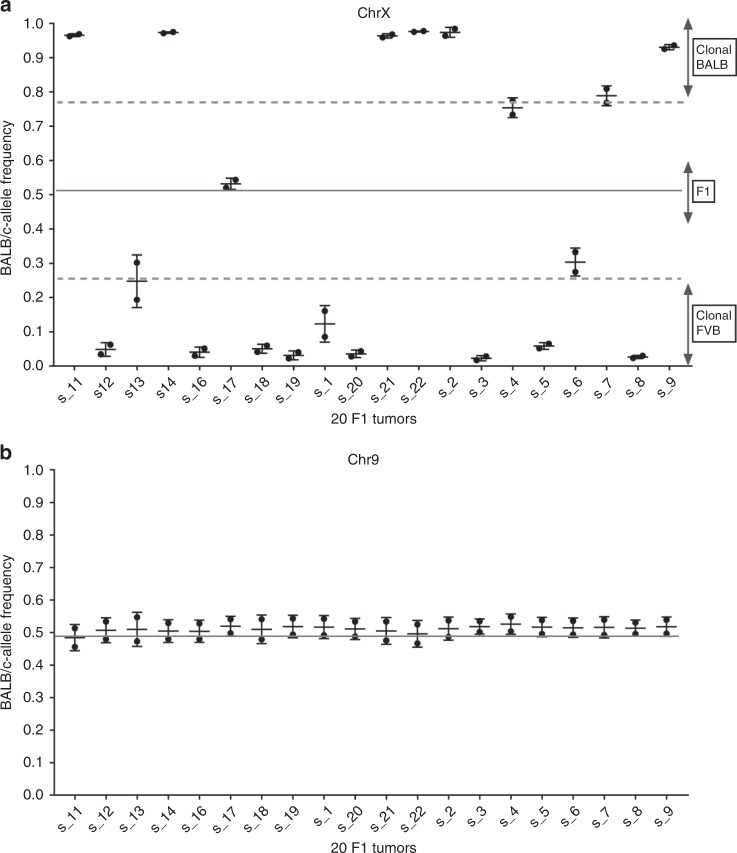
Individual tumor gene expression allelic ratios are dominated by a single ChrX demonstrating clonal origin of tumors. Locus-by-locus FVB and BALB exonic allele raw read counts were used to calculate allelic ratios ([minor allele counts] per [minor + major allele counts]). FVB allele ratios were converted to BALB allele ratios (FVB = 1-BALB) so that all eSNVs are shown as BALB/c-allelic frequencies (CAF). **a** Mean CAF are shown for eSNVs (308) on ChrX from each of 20 F1 mouse breast tumors. Individual data points represent eSNV originally quantitated as FVB and BALB. An allelic ratio of approximately 0.5 (solid line) is the expected ratio for F1 progeny. An allelic ratio of <0.25 or >0.75 is considered skewed toward one parental allele (dotted lines). **b** Mean CAF for eSNVs (446) on Chr9 as an example of the F1 nature of all autosomes

**Fig. 2:**
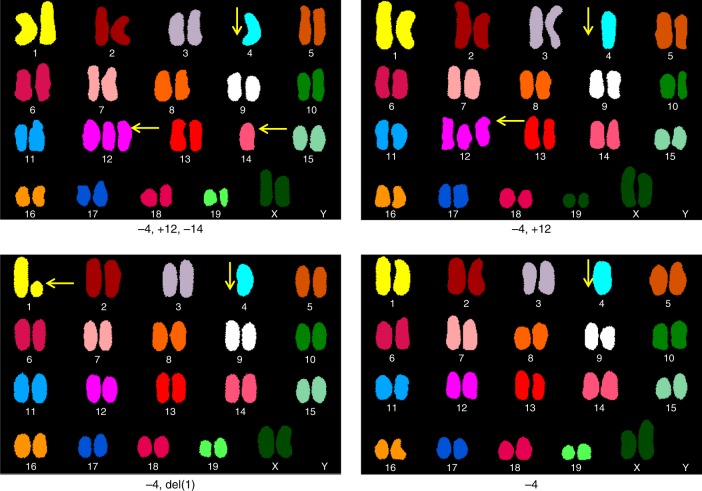
Chromosomal aberrations of F1 mouse breast tumors include a high penetrance for Chr4 loss or gain (20 out of 20) with sporadic loss or gain of chromosome material elsewhere on the genome. Primary tumor cells were cultured from 10 mouse breast tumors from 10 independent F1 mice and treated with colcemid for karyotypic analysis. Shown are typical examples (4 of 20 total) of SKYPaint® images of metaphase chromosome spreads. Arrows indicate chromosome abnormalities of Chr1, Chr12, and Chr14 reported as clonal

Consistent loss of heterozygosity (LOH) in allelic gene expression occurred in three key genomic regions. First, the majority of tumors expressed ChrX-encoding genes from either the BALB or FVB parent (Fig. 1, mean CAF <0.25 or >0.75 in 18 of 20 tumors compared to mean CAF typical of autosomes such as Chr 9 ~0.5 in 20 of 20 tumors). As X-inactivation occurs early in tissue differentiation and is transmitted to daughter cells during replication^17,18^, the quantitative dominance of one set of parental alleles indicates that these mouse tumors were clonal, each being derived from a single breast epithelial cell marked early by inactivation of one parental ChrX. The second genomic region exhibiting strong LOH was the entirety of Chr4. eSNVs all along Chr4 were highly skewed toward FVB (*P* < 10^−3^), reflecting the selective loss of BALB/c alleles (Supplementary Fig. 1). This finding is consistent with our previous DNA-based measures of LOH using SNP genotyping^13^, suggesting that this LOH is due to an imbalance in chromosome content. To confirm this, we performed karyotype analysis (Fig. 2, Table 1) of multiple F1 tumors and found that 20 of 20 tumors showed clonal loss of one copy of Chr4—a finding in common with previous reports of the instability of Chr4 in mouse tumors^6,19^. In our karyotype analyses, there were occasional clonal losses or gains of other chromosomes (particularly chromosomes 1, 12, and 14) with no apparent preference for either parental chromosome. The third loss of allelic information emerged in the eSNV expression pattern on a centromeric segment of Chr7 where 100% of the expressed alleles were FVB in origin (*P* < 10^−6^). As the BALB/c parent, whose Chr7 alleles were lost in these tumors, carries the neuT transgene and because we had observed genomic deletions associated with a transgene insertion in other contexts^20^, we considered that Chr7 of the BALB parent might be the location of the neuT insertion. We tested F1 breast tumor cells by in situ hybridization and found that a neuT probe co-localized with a marker for Chr7 on one of the sister chromosomes in cells from neuT^+^ but not neuT^−^ littermates (Supplementary Fig. 2), confirming this as the transgene integration site. Having found that RNAseq data provides meaningful evidence of genetic imbalances in allelic gene expression, we next looked more closely for stochastic imbalances in allelic expression throughout the genome.

**Table 1:** Summary of 100 mitotic images examined, focusing on dominant phenotype of Chr4 aberrations independent of clonal (83) or nonclonal losses (17)

Karyotype	[BALB/c-neuT x FVB] F1
39, XX, −4 (all or a portion)	51
39, XX, −4 and loss/gain of another(s)	49
39, XX, no chromosome changes	0

Additional nonclonal chromosome aberrations were observed for Chr1, Chr2, Chr3, Chr7, Chr8, Chr11, Chr13, Chr15, Chr16, Chr17, Chr18, and Chr19

In our previous study of heterozygous tumor DNA, we used methods directly probing native (non-amplified) DNA to measure stochastic LOH at widely dispersed loci throughout the genome^13^. This LOH was detected even when the genomic DNA sequences of both alleles were still present, an indication that apparent LOH had an epigenetic etiology. This possibility was supported by our finding that epigenetically modified DNA templates reproduced the observed distortions in allelic ratios. Remarkably, CpG, the usual target of DNA epigenetic marks was largely absent from the analyzed DNA sequences indicating that some other structural changes must be at play. The dominance of cells sharing these LOH fingerprints is most consistent with inherited DNA marks among clonally expanded cells. As known epigenetic DNA modifications alter gene expression, we assessed whether these unexpected stochastic epigenetic changes that were widespread in the DNA of developing breast tumor clones were accompanied by a corresponding stochastic pattern of imbalanced allelic ratios among the expressed genes in these tumors. To do this, we examined deviations in the expression of alleles from the characteristic underlying ratio of allelic expression evident among the study cohort of 20 breast tumors.

**Fig. 3:**
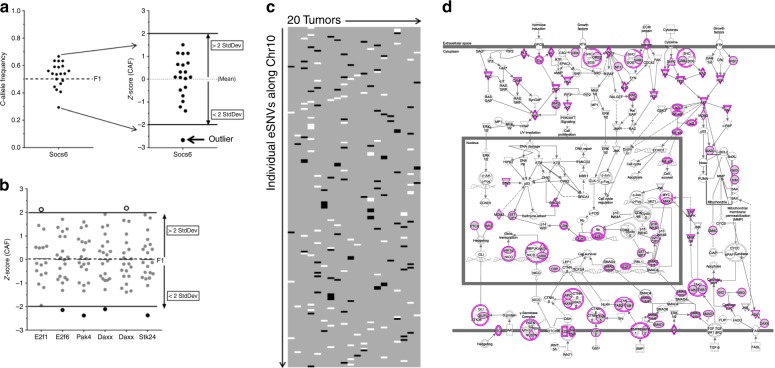
Allelic ratios of eSNVs in F1 tumors have a high proportion of single-tumor outliers which are distributed in a variegated pattern across the genome (V-LOH) and enriched for genes whose dysregulation define the cancerous state. **a** Example of method to convert CAF to Z-score (=(X-μ) σ^−1^; where X = CAF, μ = mean; σ = StdDev) for each tumor sample for the eSNV within the gene *Socs6*. A tumor having a *Z*-score >2 or <−2 is defined as an outlier. In this example, the outlier has a Z-score <−2 and is thus shown as a black circle. **b** Expanded examples of loci in which an individual tumor expresses an eSNV outlier,shown as a white (open) circle (*Z*-score > 2), or a black circle (*Z*-score < −2), in a field of otherwise gray circles representing a normal distribution of allelic ratios present in the other 19 tumors. **c** Coloration scheme as in (**b**) for all eSNV across Chr10. Outliers appear in a variegated (V-LOH) pattern. In addition to the tumors expressing a normal eSNV ratio, those linked eSNVs which were skewed toward one parent and are interpreted by the authors to represent chromosome loss (see ref. ^13^) and removed from this analysis, are also shaded gray. **d** The genes corresponding to all eSNVs having a single outlier (Supplementary Fig. 3, Supplementary Data) were analyzed by Ingenuity Pathway Analysis (IPA). Shown is the IPA canonical pathway, Molecular Mechanisms of Cancer (*p* = 3.02 E-08, Fisher’s exact test adjusted for multiple hypothesis testing), the top scoring pathway. This *p*-value from IPA is related to the probability that the association between the genes in the dataset and the canonical pathway is explained by chance alone^21^. Gene products in color represent the 90 genes expressed in F1 tumors mapping to this pathway (see Supplementary Table 1)

The analysis focused on testing whether, for each eSNV, an individual tumor had an aberrant ratio of expressed alleles compared to the other tumors in the cohort. We first corrected for DNA-based allelic imbalances by excluding genes on Chr4, ChrX and the centromeric end of Chr7 where all tumors had skewed allelic ratios. We found the CAF of the remaining eSNV (11,220) to be normally distributed, with an average mean CAF across these eSNV to be 0.511 ± 0.096. We then tested each eSNV for the occurrence of an allelic ratio outlier outside the distribution of allelic ratios described by the 20 tumors. To do this, an individual *Z*-score (*Z* = (value – mean) (std dev)^−1^) for the expression ratio for each tumor at every eSNV was calculated, and using a threshold Z-score of 2 a pattern of outliers was revealed (Fig. 3a–c, Supplementary Fig. 3). We found allelic ratio outliers both above (positive *Z*-score) and below the mean allelic ratios (negative *Z*-score), and outliers were present in genes throughout the tumor genomes. Moreover, the pattern of observed outliers was variegated, much like that observed at the DNA level (V-LOH)^13^. Based on the expected distribution of outliers in 20 samples, the frequency of tumors with one or more outliers (~63%) should follow the Poisson distribution. We found, however, 87% (2634 of 3044) of expressed genes marked by eSNVs were represented in at least one tumor at an allelic frequency greater than 2 STD DEV away from the empirical mean of the tumor population (*p* < 10^−6^). This strong bias in favor of an excessive number of outliers indicates that, during tumor development, selection favors cells with these minor changes in gene expression.

The nature of genes in which we observed stochastic allelic imbalances (i.e., V-LOH, Supplementary Data) was explored further using pathway analysis and found to be significantly enriched in pathways that define the cancerous state (Molecular Mechanisms of Cancer (Fig. 3d), mTOR signaling, Telomerase Signaling, and others, IPA analyses^21^, Qiagen; Supplementary Table 1). Gene Ontology (GO) enrichment analyses^22–24^ of this same gene set identified a similar enrichment in pathways regulating cancer traits (Panther Pathways^23^: p53 Signaling, Angiogenesis Signaling, *P* < 0.01 for both pathways). A comparison of our gene set with that from a recent transcriptome-based search for new breast cancer susceptibility genes^7^ found fourteen exact matches (shown in bold, Supplementary Table 1) and more than thirty that match highly related genes. As an independent check on the power of our approach, we analyzed ten random gene sets and found no such enrichment of pathways using either IPA or GO analyses. The consortium of molecules comprising the Molecular Mechanisms of Cancer dominates pathways involved in growth factor activated signalling, evasion of cell death and senescence, sustained angiogenesis, and limitless replicative control (Fig. 3d). In essence, the pathways identified in our analysis function normally to maintain homeostasis and are identified as cancer-associated when homeostasis is lost.

A second and non-overlapping pathway, Antigen Presentation (*P* = 2.62 E-04, Supplementary Table 1), also emerged in our analysis of the allelic ratio outliers within our breast cancer tumors. The biological importance of this pathway to tumor development is reinforced by the observation that as the *Z* score threshold defining outliers was increased, the prominence of this pathway in the analysis increased (*P* = 2.62 E-04 using a *Z*-score threshold of 2.5; *P* = 2.95 E-02 for Molecular Mechanisms of Cancer, see Supplementary Table 2). The interplay between evolving cancers and the immune system has been documented extensively^25^. One implication of this finding is that individual tumors devise independent strategies to alter their interaction with elements of the immune response. The full nature of this heterogeneity due to changes in gene expression defined by the altered eSNV ratios remains to be determined.

**Fig. 4:**
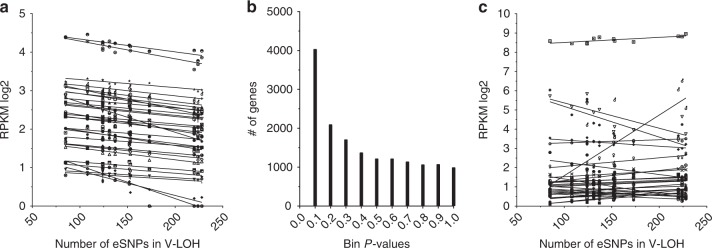
Individual F1 tumors vary in the number of allelic ratio outliers, and that frequency negatively correlates with the expression levels of many genes, demonstrating genome-wide changes in transcription regulatory processes. The number of eSNVs whose absolute *Z*-score exceeded 2 were enumerated for each of 12 tumors that had no evidence of concerted LOH. That number of allelic ratio outliers was then entered into a correlation analysis using all genes expressed at least 0.5 RPKM log2 in those same tumors. In (**a**) correlations are depicted for the top 30 genes for ease of viewing (for complete listing, see Supplementary Data). All correlations shown had a *P*-value < 0.003 and a FDR < 0.15. **b** Distribution of *P*-values is shown in bins from 0.1 to 1.0. **c** A typical lack of correlation (one of ten tests, with similar results) conducted by randomizing the order of the tumors in the dataset

Mechanistic insights followed from the observation that the penetrance of V-LOH across these mouse breast tumors was variable among the tumor samples. We evaluated whether a relationship existed between the frequency of V-LOH in gene expression in individual tumors and the overall expression of any specific genes in those cancer cells (Fig. 4a, b). The expression levels of 748 of 15,975 genes displayed strong correlations (based on an absolute *R* > 0.7 and *P* < 0.01 threshold; Supplementary Data) that were not found using random gene sets (Fig. 4c). Assuming a frequency of 1% of genes randomly displaying a correlation of this magnitude (i.e., ~160 genes), the observed frequency of 4.7% is a highly enriched frequency of correlated genes (*P* < 10^−6^, binomial probability test). Furthermore, 98.6% of those correlations with *P* < 10^−6^ (Supplementary Table 2) were negative, indicating that the mechanism at play involves widespread repression of RNA transcription or stability. Indeed, GO analysis using the top 100 of these genes found a 4.88-fold enrichment for genes participating in Negative Regulation of Transcription by RNA Polymerase II (FDR = 2.27 E-03). This pattern is also consistent with previous reports documenting down-regulation of genes by epigenetic mechanism in cancers^26^. How the mechanisms down regulating genes and altering the expressed ratios of alleles relate to each other is not known, but their high correlation suggests that mechanisms operating at the level of DNA^13^ to suppress gene expression of cis-linked transcripts could also account for depression of just one allele if it operates stochastically throughout the genome. Our findings suggest that these minor alterations in multiple genes, representing multiple pathways are selected during the evolution of these breast cancers. Interestingly, the two most strongly correlated genes, *Etv3* and *Zfp217*, are transcription regulators shown by others to be associated with breast cancers^27,28^, further suggesting that widespread transcriptional dysregulation promotes diversity in cancer phenotypes.

Using an F1 spontaneous cancer model we show in Her2/neu-driven tumors that allelic ratio outliers were present in F1 tumors at a higher than expected frequency and were variably distributed across the genomes of the tumors studied, the sampled tumors were dominated by single clones, the genes containing eSNV outliers were enriched in pathways that define homeostatic and antigen presentation pathways and whose dysregulation often define the cancerous state (rapid cell growth and proliferation, immune evasion), and there was an overwhelming bias toward a negative correlation of gene expression with the observed frequency of outlier eSNV ratios in developed tumors. In a previous study of F1 tumors, we found evidence of epigenetic (i.e., non-genetic) processes capable of causing random distortions in allelic ratio measurements^13^. We take the observations presented here as evidence of sporadic errors in epigenetic regulatory mechanisms that promote repression of cis-linked transcripts and that this genome-wide molecular chaos is a foundation for heritable tumor diversity. However, the relationship between the events at the DNA level and those mechanisms regulating gene expression remains undefined.

The impact of cis- and transacting variability in epigenetic regulation in normal tissues has been suggested by transcriptome data from smaller studies of normal tissues from F1 heterozygotes^10,11,29^. Our own analysis of data reported by Keane et al.^29^ found that the variation of allelic ratios expressed in those tissues was actually distributed more broadly than in the clonal tumors studied here, a finding consistent with their conclusion that allelic ratios of expressed genes are different in different tissues. We were unable to discern outlier patterns in the normal tissue data due to limited sample sizes. However, an IPA analysis of genes marked by potential outlier eSNV in the data sets derived from normal liver and normal hippocampus did not reveal significant enrichment of genes associated with cancer or antigen presentation, while an identically powered analysis of samples from our tumor cohort recapitulated the cancer profiles reported above (*P* = 0.0002, corrected for multiple hypothesis testing). There thus exists a certain propensity for transcript variability in normal, homeostatic mechanisms that becomes enriched during tumorigenesis.

In recent studies of human lymphocytes^8^, we demonstrated that small changes in gene expression, when integrated across multiple genes, were descriptive of individual response phenotypes to subsequent immune activation. In a similar vein, this study reiterates this principle by demonstrating that small changes in gene expression among combinations of genes whose normal functions are to maintain homeostasis are selectively enriched and therefore must determine differential responses of expanding tumor clones to challenges and opportunities present within the microenvironment.

## Methods

### Mice

These animal studies were conducted using protocols approved by the Mayo Clinic Institutional Animal Care and Use Committee (IACUC). Hemizygous BALB/c-neuT mice were originally acquired from Dr. Guido Forni and were maintained by intercross with transgene negative BALB/c female as described previously^13^. F1 hybrid mice (female progeny only) were from matings of neuT transgene positive BALB/c males with wildtype FVB/J females and were studied according to IACUC protocol #A47311-11. Animals were allowed to develop spontaneous breast cancer tumors (generally at 14–20 weeks), and monitored weekly for the appearance of palpable tumors at which point they were monitored daily. All animals developed multiple tumors (≥5 of 10 glands affected). When any tumor reached 100 mm^2^ (width × length), the mouse was killed using CO_2_ inhalation and one tumor removed post mortem and stored frozen at −80 C until processing for RNA.

### RNA samples and RNAseq

RNA was isolated from small pieces (<50 mg wet weight) of frozen breast tumors which were thawed, weighed and immediately homogenized using a Tissue Miser (Fisher), miRNeasy kit followed by DNase treatment, and additional clean-up using RNeasy MinElute Cleanup (all Qiagen). All further steps for RNAseq were conducted by the Mayo Clinic Gene Expression Core, including TruSeq library preparation and Paired-end (PE)150 analysis using HiSeq4000, 8 samples per lane (Illumina), data processing using MapRseq 2.0.0^30^, read alignment using TopHat v2 using Mouse Genome Build, mm10 and primary data FastQC. While the initial run yielded reads of up to 150 bases, all sample reads were trimmed to 100 bases for downstream analyses to enhance the quality of the data.

### eSNV analyses and statistics

Germline-encoded sequence variants of FVB/J and BALB/c mouse strains were downloaded from the Mouse Genome Project (http://www.sanger.ac.uk/science/data/mouse-genomes-project). Unique variants specific to FVB and BALB were extracted and the read depth of both FVB alleles and BALB alleles were counted in each.bam file using SAMtools^31^. The FVB and BALB allele counts for 56,328 unique loci were then copied into an Excel spreadsheet (Microsoft Office 2010) for further analyses. To keep our focus on genes consistently expressed with good exon coverage across samples, we kept only eSNVs where the minor allele count was at least 15. We then calculated allelic ratios for BALB as [BALB allele counts] per [(FVB + BALB) allele counts]. FVB allele ratios were calculated in the same way, then converted to BALB allele ratios (FVB = 1-BALB). Thus, all eSNVs are considered using BALB/c-allelic frequencies (CAF). To compare CAF variability across tumor samples, we calculated *Z*-scores at each polymorphic locus using the mean and standard deviation (std dev) for the 20 tumors (*Z* = (value − mean) std dev^−1^). CAF data for 50 eSNV were arbitrarily sampled and tested for normality using Prism 7 (GraphPad). In that test, 92% of the eSNV tested passed using the optional Shapiro Wilk test^32^; of note, all sampled eSNV passed this test when any individual tumor sample containing outliers were removed from the analysis during the characterization of V-LOH.

LOH was characterized in two ways: first as eSNV in which all tumors displayed allelic loss (mean CAF <0.25 or >0.75), and secondly as eSNV in which the allelic ratio for at least one tumor was more than 2 std dev away from the mean for that locus. After removal of eSNV found to display allelic loss (e.g., all of Chr4 and ChrX, part of Chr7), outliers were defined as having a *Z* score of ≥2 or ≤−2; conditional formatting tools in Excel were used to visualize each cell (black fill for *Z* ≤ −2, gray fill for *Z* between −2 and 2, white fill for *Z* ≥ 2). As 20 tumors were evaluated, a random frequency of outlier measures was expected to be 1 in 20. Using the Poison distribution, an expectation of allelic-frequency measures among the 20 tumors for each eSNV-marked gene containing no outliers was estimated to be 0.3679. Using these expected values, the probability of the observed frequencies of eSNVs with no outliers was calculated using the binomial distribution.

eSNV containing outliers were pooled to compile a gene set for further characterization. Correlations of gene expression and outlier frequency in individual tumors were performed using the ANOVA option in Partek Genomics Suite 6.6. Random data sets were generated using Excel. Characterization and statistical analyses of derived gene sets were conducted using IPA^21^ with the optional Benjamini-Hochberg correction for multiple hypothesis testing^33^. The online tools (http://geneontology.org/) of the Gene Ontology Consortium (GO^22,24^) were also employed to characterize the gene sets. All graphs were generated using Prism.

### Statistics and reproducibility

The data presented were derived from a single study of 20 independent F1 mouse tumors, 1 tumor from each of 20 mice bearing multiple tumors at the time of sampling. RNA from these tumors were processed and sequenced at the same time in order to minimize batch effects. The raw data are the result of the interrogation of thousands of genomic loci for each of the samples in a single data set which has been deposited in the GEO database. All data to prepare the Figures are reported in the main text or in Supplementary Information. In summary, calculations of CAF (representing the relative expression of each of two parental alleles) used eSNV counts of at least 15 raw counts in order to focus on genes consistently expressed with good exon coverage across all samples. To test for normality in the data, CAF data were arbitrarily sampled and tested using the optional Shapiro Wilk^32^ test in Prism 7 (GraphPad). Much of the analysis focuses on the assumption of equal chance for each parental allele to be expressed for most genes in each of these F1 tumors. We examined these allelic ratios across thousands of eSNV, representing hundreds of expressed genes. It was our assumption that the expected distribution of outliers (an allelic ratio for a given eSNV being greater than 2 standard deviations outside the mean for 20 samples) should follow the Poisson distribution. To test for allelic ratio outliers irrespective of mean, CAF were converted to *Z*-scores; eSNV in which only 1 in 20 contained an outlier ratio were explored further using Students’ t-test and binomial distribution. Correlations of gene expression and outlier frequency in individual tumors were performed using the ANOVA option in Partek Genomics Suite 6.6. Characterization and statistical analyses of derived gene sets were conducted using Ingenuity Pathway Analysis (IPA^21^) with the optional Benjamini-Hochberg correction for multiple hypothesis testing^33^. As independent checks on the power of these pathway analyses, we generated random gene sets using Excel. Correlation tests used ANOVA option in Partek Genomics Suite 6.6.

### Spectral karyotype (SKY) and in situ hybridization

SKY karyotype and DNA fluorescence in situ hybridization (FISH) experiments were performed by the Mayo Clinic Cytogenetics Core using primary cultures derived from F1 mouse breast tumors and freshly prepared normal fibroblasts. Briefly, fresh tumors were mechanically dissociated and cultured in RPMI 10% FBS containing Penn-Strep for 24–48 h, any debris was removed, the media replaced, and adherent cells were cultured for 1–3 weeks until sufficiently confluent. Cells were stored in liquid nitrogen until they could be processed for SKY. Reagents for SKY were from Applied Spectral Imaging. SKY images were captured using an Axioplan 2 microscope (Zeiss) and GenASis software version 7.27 (Applied Spectral Imaging). FISH images were captured using an Axioplan 2 microscope (Zeiss) and CytoVision Imaging software version 7.4 (Leica). The mouse centromeric probe BAC RP23-209m4 located at 7qA2 was provided by the BACPAC Resource Center (https://bacpacresources.org/) at Children’s Hospital Oakland Research Institute (Oakland, CA) and used to test the hypothesis that the transgene integration site was adjacent. The plasmid pSV2neuT plasmid was obtained from Addgene (http://www.addgene.org/) and was a gift from Bob Weinberg^34,35^.

### Reporting summary

Further information on research design is available in the Nature Research Reporting Summary linked to this article.

## Supplementary information

Supplementary Information

Description of Additional Supplementary Items

Reporting Summary

Supplementary Data

## Data Availability

The data for this study have been deposited in the NCBI Gene Expression Omnibus (GEO) database under the GEO accession number GSE128775.
