# Supplementary material for: Stochastic changes in gene expression promote chaotic dysregulation of homeostasis in clonal breast tumors
Source: Commun Biol. 2019 Jun 14;2:206. doi: 10.1038/s42003-019-0460-0 (PMC6570763; doi:10.1038/s42003-019-0460-0)
Supplement: Supplementary file 1 — Supplementary Information [file 42003_2019_460_MOESM1_ESM.pdf]

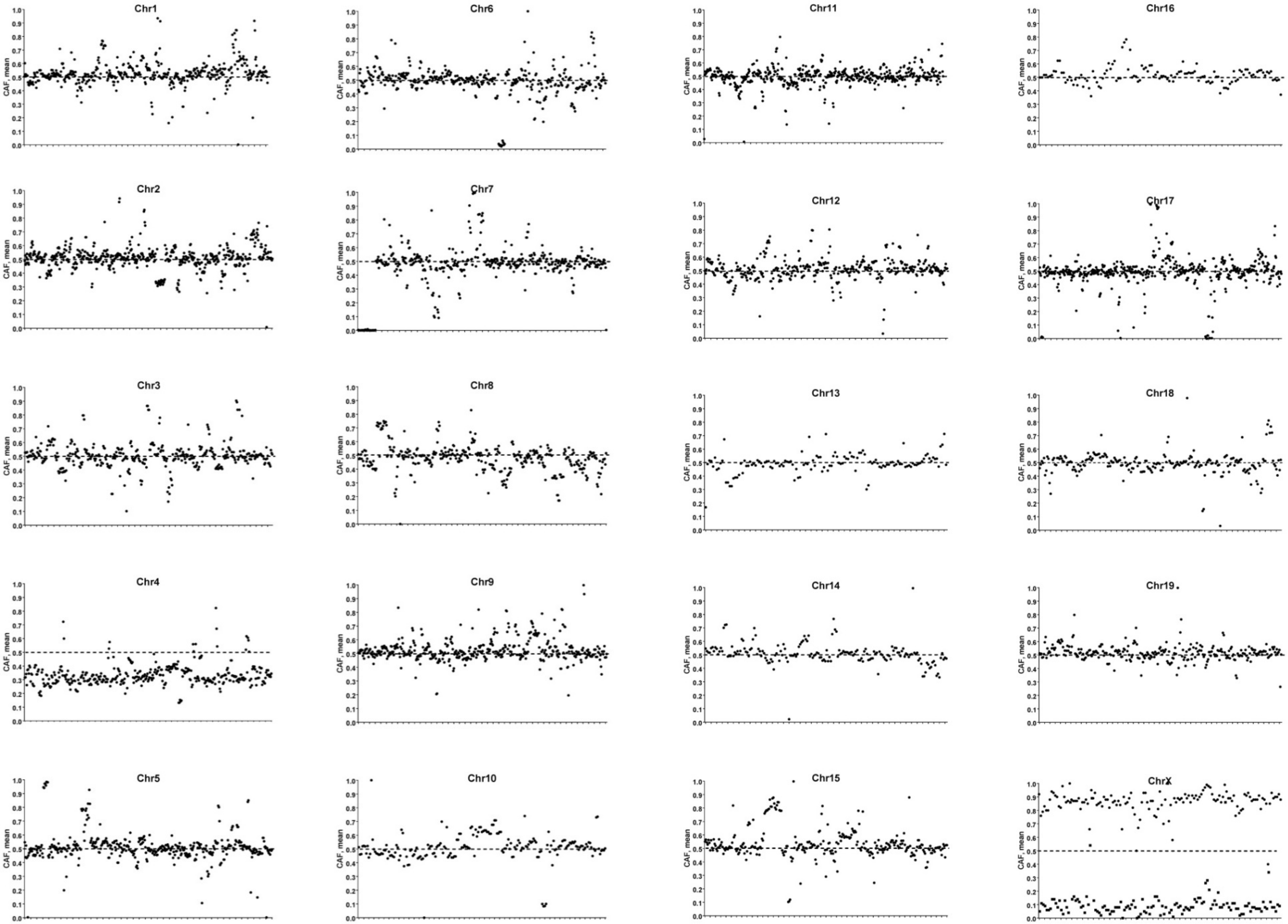

Supplementary Figure 1. Allelic gene expression in F1 mouse breast tumors. Allelic ratios, presented as BALB/c-allele frequency (CAF), were calculated from raw read counts for ~12,400 expressed polymorphisms (expressed single nucleotide variants, eSNV). Mean CAFs for 20 individual tumors (1 per mouse) are shown for each of the 20 chromosomes.

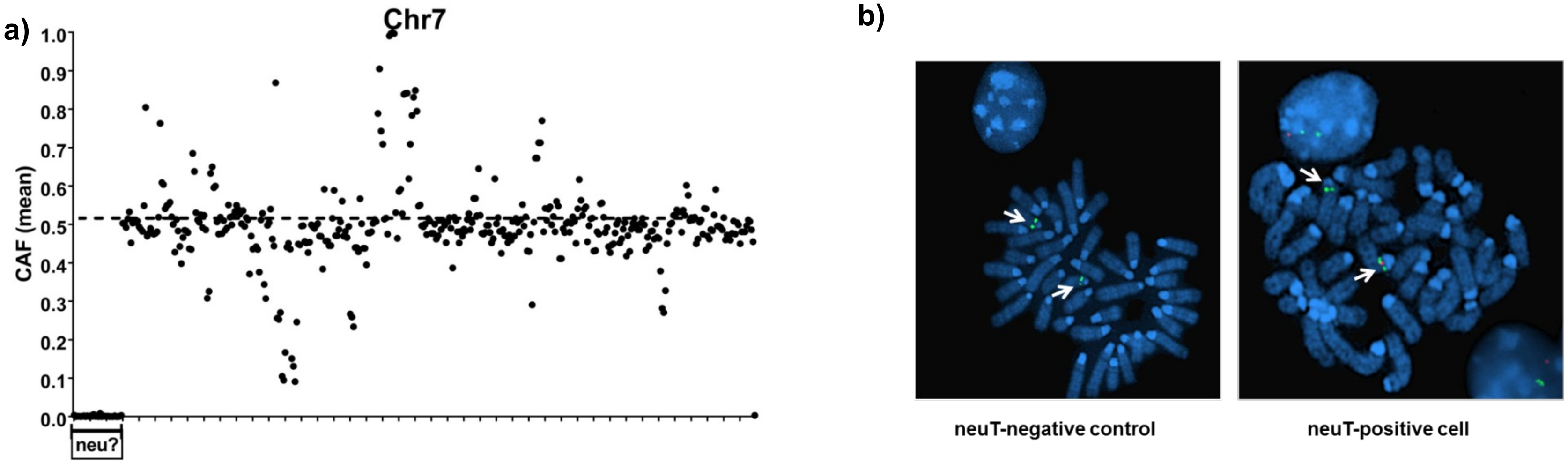

Supplementary Figure 2. neuT transgene localized onto proximal arm of mouse chromosome 7. a) Enlargement from Supplementary Figure 1 of Chr7 showing mean CAF at ~ zero, indicating that all eSNVs are encoded by the FVB parental chromosome); b) visualization of neu^{-} and neu^{+} F1 mouse tissue chromosomes by FISH. Green = Chr7 centromere probe. Red = Rat/neuT-specific probe.

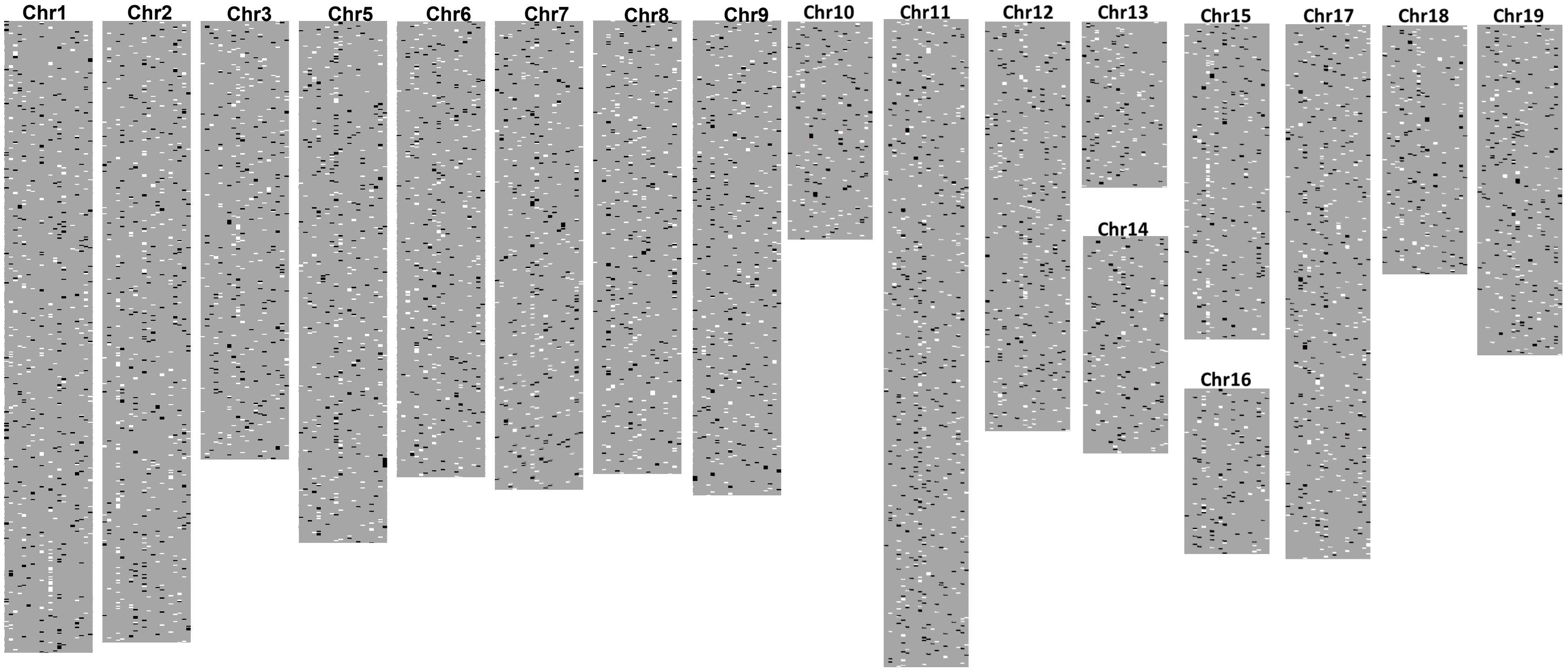

Supplementary Figure 3. Allelic ratios of eSNVs in F1 tumors are distributed in a variegated pattern across the genome (V-LOH). Shown are 6344 of 11363 eSNVs (corresponding to 2389 genes) across the 19 autosomes for whom the allelic ratio of one in 20 tumors is an outlier (see Fig. 3). Individual CAF are shaded as white (Z-score > 2) or black (Z-score < -2), or otherwise gray. Those linked eSNVs which were skewed toward one parent and are interpreted by the authors to represent chromosome loss (see text and Ref[1]) and removed from this analysis, are also shaded gray.

SUPPLEMENTARY TABLE 1

**Ingenuity Canonical Pathways**	**B-H p-value**	**-log(B-H p-value)**	**Molecules**
Molecular Mechanisms of Cancer	3.01995E-08	7.52E+00	PRKACB,BAD,AXIN1,SMAD3,TAB2,NCSTN,MAPK13,E2F6,ITGA3,RHOB,SUFU,PIK3CG,ARHGEF11,GNA13,NFKBIB,SMAD1,FZD2,WNT5B,CDC25A,SMAD2,CDK13,AKT2,CREBBP,TYK2,PTCH1,RHOJ,NFKB2,AURKA,APC,DAXX,CDH1,MAX,RHOQ,PTPN11,RND3,IRS1,E2F1,PRKACA,FZD6,PLCB3,ARHGEF18,MAP2K3,FZD5,GNAL,CAMK2G,PIK3CA,RALA,LRP6,BMPR2,CRK,FZD1,SMAD5,GNA14,MAP3K5,HIF1A,NFKB1,CDK10,EP300,DIABLO,CASP6,AKT1,RHOD,PIK3C3,BID,AKT3,ARHGEF2,CASP8,PRKCA,LRP5,PAK4,ARHGEF12,PIK3C2A,HAT1,GRB2,GNAQ,SMAD7,MDM2,SIN3A,ARHGEF5,CDKN2D,PRKAR2B,MAPK14,RRAS2,NF1,CDKN1A,CDKN1B,ADCY7,BCL2L11,CTNND1,FZD7
PPARα/RXRα Activation	4.46684E-05	4.35E+00	PRKACB,GPD1,RALA,PRKAB1,PRKAB2,SMAD3,TGFBR3,BMPR2,GNA14,NFKB1,EP300,HSP90B1,HSP90AB1,IL1RL2,CLOCK,NFKBIB,GOT2,PRKCA,SMAD2,MAP2K7,MED1,GRB2,ACOX1,CREBBP,GNAQ,ACVR1,NR2C2,BCL3,NFKB2,NCOA3,TRAF6,ACADL,MAPK14,PRKAR2B,GHR,RRAS2,IRS1,PRKACA,PLCB3,IL1B,MAP2K3,NCOR2,INSR,ADIPOR2,MAP4K4,ADCY7
mTOR Signaling	4.46684E-05	4.35E+00	PRKAB1,PRKAB2,PPP2R5B,PDPK1,RICTOR,VEGFA,RHOB,PIK3CG,TSC2,AKT1S1,AKT2,RHOJ,EIF3E,MLST8,EIF3M,PPP2R1A,RHOQ,PTPN11,RND3,PPP2R3A,PTPA,EIF4A3,IRS1,RPS25,INSR,EIF3K,PIK3CA,RALA,RPS18,HIF1A,PDGFC,AKT1,RHOD,PIK3C3,AKT3,PPP2R2C,PRKCA,MAPKAP1,PIK3C2A,GRB2,FAU,PLD4,EIF3G,RRAS2,RPS27L,PRR5,RPS6KA4,PPP2R5E,PPP2R1B,ARHGAP8/PRR5-ARHGAP8
Germ Cell-Sertoli Cell Junction Signaling	0.000114815	3.94E+00	EPN3,PIK3CA,MAP3K11,RALA,AXIN1,PDPK1,MAP3K5,TUBB,BCAR1,ITGA3,AKT1,RHOB,RHOD,KEAP1,PIK3CG,PIK3C3,TUBA1C,VCL,ACTA1,RAB8B,ACTN1,PAK4,PXN,MAP2K7,PIK3C2A,GRB2,RHOJ,EPN2,MAP3K12,CDH1,MAPK14,RHOQ,TUBA1A,RRAS2,TUBB6,PTPN11,RND3,IRS1,MAP2K3,ACTN4,CLINT1,CTNND1,NECTIN2
Sertoli Cell-Sertoli Cell Junction Signaling	0.000114815	3.94E+00	EPN3,PRKACB,SPTBN1,MAP3K11,RALA,AXIN1,TGFBR3,SYMPK,MAP3K5,MAPK13,TUBB,BCAR1,ITGA3,TJAP1,AKT1,KEAP1,CGN,SPTB,AKT3,NECTIN1,TUBA1C,VCL,ACTN1,ACTA1,RAB8B,SPTBN2,MAP2K7,AKT2,TJP2,YBX3,F11R,EPN2,MAP3K12,CDH1,MAPK14,PRKAR2B,TUBA1A,RRAS2,TUBB6,PRKACA,MAP2K3,ACTN4,CLINT1,NECTIN2
Telomerase Signaling	0.000114815	3.94E+00	PIK3CA,RALA,PPP2R5B,TERF2IP,HDAC10,PDPK1,HSP90B1,AKT1,SP1,HSP90AB1,PIK3C3,PIK3CG,AKT3,PPP2R2C,EGFR,ETS1,AKT2,HDAC4,PIK3C2A,GRB2,HDAC5,ELF2,PPP2R1A,RRAS2,PTP

			N11,PPP2R3A,PTPA,IRS1,CDKN1A,E2F1,PPP2R5E,PPP2R1B
Adipogenesis pathway	0.000114815	3.94E+00	DGKD,SMAD3,SAP30L,HDAC10,FBXW7,PPIP5K1,BMPR2,FZD1,SMAD5,HIF1A,AKT1,LPIN1,CTBP2,CEBPA,CLOCK,GTF2H5,SMAD1,FZD2,KMT2B,GTF2H3,HDAC4,HAT1,TXNIP,XBP1,SAP130,SIN3A,HDAC5,Kat6b,SIRT2,GTF2H4,SREBF1,FZD6,FZD5,SOX9,FZD7
Wnt/β-catenin Signaling	0.000114815	3.94E+00	CSNK1G1,AXIN1,TGFBR3,SOX10,LRP6,PPP2R5B,MARK2,CSNK1A1,BMPR2,FZD1,KREMEN1,SOX13,EP300,AKT1,AKT3,PPP2R2C,CSNK2B,FZD2,WNT5B,SOX5,AKT2,LRP5,CSNK1G3,CREBBP,CSNK1D,GNAQ,ACVR1,MDM2,APC,PPP2R1A,CDH1,DVL2,PTPA,PPP2R3A,FZD6,CD44,FZD5,SOX9,PPP2R5E,PPP2R1B,TCF7L2,FZD7
PI3K/AKT Signaling	0.000151356	3.82E+00	PIK3CA,RALA,BAD,PPP2R5B,PDPK1,MAP3K5,NFKB1,YWHAQ,ITGA3,HSP90B1,AKT1,HSP90AB1,PIK3CG,TSC2,AKT3,PPP2R2C,NFKBIB,AKT2,CDC37,YWHAE,GRB2,TYK2,MDM2,NFKB2,SYNJ2,PPP2R1A,RRAS2,INPP5F,PTPA,PPP2R3A,CDKN1A,PPP2R5E,CDKN1B,PPP2R1B
Xenobiotic Metabolism Signaling	0.000151356	3.82E+00	CAMK1,MAP3K11,PPP2R5B,MAPK13,ARNT,CHST2,KEAP1,PIK3CG,MGST1,HDAC4,MED1,CREBBP,SNW1,NFKB2,HDAC5,HS3ST3B1,MAP3K12,PPP2R1A,PTPN11,PPP2R3A,PTPA,IRS1,MAP2K3,ALDH3B1,ALDH16A1,NDST1,CAMK2G,PIK3CA,RALA,GSTM5,NQO2,HS2ST1,MAP3K5,NFKB1,SOD3,HS3ST3A1,EP300,ALDH2,HSP90B1,HSP90AB1,ALDH1A3,PIK3C3,PPP2R2C,NFE2L2,CITED2,PRKCA,GSTM1,SRA1,MAP2K7,PIK3C2A,GRB2,UGT8,FTL,RRAS2,MAPK14,CAT,IL1B,NCOR2,PPP2R5E,PPP2R1B,DNAJC7
Tight Junction Signaling	0.000169824	3.77E+00	PRKACB,CPSF2,PPP2R5B,MARK2,MPP5,SYMPK,NFKB1,VTI1B,CPSF6,AKT1,LLGL1,CGN,CEBPA,AKT3,ARHGEF2,PPP2R2C,NECTIN1,SAFB,VCL,NAPA,ACTA1,AKT2,CSTF1,TJP2,YKT6,MYH14,RAB13,YBX3,NFKB2,F11R,PPP2R1A,PRKAR2B,PTPA,PPP2R3A,PRKACA,CPSF3,PPP2R5E,CSTF3,PPP2R1B,NECTIN2
Epithelial Adherens Junction Signaling	0.000169824	3.77E+00	EPN3,RALA,TGFBR3,BMPR2,CRK,TUBB,CLIP1,NOTCH2,AKT1,KEAP1,AKT3,NECTIN1,TUBA1C,VCL,ACTA1,ACTN1,EGFR,DLL1,AKT2,NOTCH3,MYH14,ACVR1,APC,MET,EPN2,CDH1,NOTCH4,TUBA1A,RRAS2,TUBB6,SSX2IP,ACTN4,PARD3,CLINT1,TCF7L2,CTNND1,NECTIN2
Breast Cancer Regulation by Stathmin1	0.00018197	3.74E+00	PRKACB,PIK3CA,Calm1 (includes others),CAMK1,RALA,PPP2R5B,TUBB,ROCK2,E2F6,PPP1R10,PIK3C3,PIK3CG,ARHGEF2,PPP2R2C,TUBA1C,ARHGEF11,GNA13,PRKCA,ARHGEF12,PIK3C2A,GRB2,ITPR2,GNG2,GNAQ,PPP1R11,ITPR1,ARHGEF5,PPP2R1A,PRKAR2B,TUBA1A,RRAS2,TUBB6,PTPN11,PTPA,PPP2R3A,IRS1,E2F1,CDKN1A,PRKACA,PLCB3,UHMK1,ARHGEF18,PPP2R5E,CDKN1B,PPP2R1B,ADCY7,CAMK2G
Sumoylation	0.000218776	3.66E+00	ARHGDIG,RAN,MAP3K5,NFKB1,NR3C1,EP300,S

Pathway			P1,RHOB,RHOD,CTBP2,RANGAP1,RFC2,CEBPA,ETS1,CREBBP,MDM2,RHOJ,NFKB2,RFC5,ARHGDIB,DAXX,RNF4,CDH1,RHOQ,RND3,PML,RFC3
Pancreatic Adenocarcinoma Signaling	0.000234423	3.63E+00	PIK3CA,RALA,BAD,SMAD3,NFKB1,PDGFC,VEGFA,RAD51,E2F6,AKT1,PIK3C3,PIK3CG,AKT3,ERBB2,EGFR,SMAD2,AKT2,PIK3C2A,GRB2,TYK2,HBEGF,MDM2,NFKB2,SIN3A,PLD4,PTPN11,IRS1,CDKN1A,E2F1,TGFA,CDKN1B
ILK Signaling	0.000234423	3.63E+00	FLNB,PIK3CA,PPP2R5B,PDPK1,HIF1A,RICTOR,NFKB1,PDGFC,EP300,VEGFA,NCK2,AKT1,RHOB,RHOD,PIK3CG,PIK3C3,AKT3,PPP2R2C,VCL,ACTA1,ACTN1,DSP,PARVA,AKT2,PXN,PIK3C2A,GRB2,MYH14,CREBBP,RHOJ,NFKB2,CREB3L4,PPP2R1A,CDH1,RHOQ,PTPN11,RND3,PTPA,PPP2R3A,IRS1,RPS6KA4,PPP2R5E,ACTN4,PPP2R1B
HIPPO signaling	0.000234423	3.63E+00	WWTR1,SMAD3,PPP2R5B,NF2,FRMD6,SMAD5,YWHAQ,TEAD1,PPP1R10,LLGL1,PPP2R2C,SMAD1,SMAD2,TJP2,YWHAE,CSNK1D,PPP1R11,PPP2R1A,PTPA,PPP2R3A,WWC1,CD44,PPP2R5E,PARD3,PPP2R1B
Unfolded protein response	0.000234423	3.63E+00	CALR,SCAP,MAP2K7,Hspa1b,HSPA1A/HSPA1B,XBP1,OS9,ATF6,MAP3K5,HSPA2,EIF2A,SEL1L,HSP90B1,PDIA6,SREBF1,SREBF2,EDEM1,CEBPA,NFE2L2
Antigen Presentation Pathway	0.000234423	3.63E+00	B2M,PSMB9,CALR,HLA-A,HLA-DQA1,PSMB8,CD74,HLA-DQB1,TAP1,HLA-DMA,HLA-DRA,TAP2,TAPBP,HLA-E,HLA-DRB5
Mouse Embryonic Stem Cell Pluripotency	0.000416869	3.38E+00	IL6ST,PIK3CA,RALA,AXIN1,BMPR2,FZD1,SMAD5,MAPK13,AKT1,PIK3CG,PIK3C3,AKT3,SMAD1,FZD2,AKT2,PIK3C2A,GRB2,CREBBP,TYK2,APC,DVL2,MAPK14,RRAS2,PTPN11,IRS1,FZD6,FZD5,TCF7L2,FZD7
RAR Activation	0.000436516	3.36E+00	PRKACB,PIK3CA,SMAD3,PDPK1,MAP3K5,SMAD5,MAPK13,NFKB1,SMARCA4,EP300,VEGFA,AKT1,ALDH1A3,PIK3CG,GTF2H5,AKT3,CSNK2B,RXRB,SMAD1,CITED2,PRKCA,GTF2H3,SMAD2,SRA1,AKT2,MED1,CREBBP,SMAD7,SNW1,NFKB2,MAPK14,PRKAR2B,SMARCA2,GTF2H4,IGFBP3,PRKACA,NCOR2,SMARCC1,PML,CRABP2,ADCY7,CARM1
Prostate Cancer Signaling	0.00057544	3.24E+00	PIK3CA,RALA,BAD,PDPK1,NFKB1,EP300,HSP90B1,AKT1,HSP90AB1,PIK3CG,PIK3C3,AKT3,NFKBIB,AKT2,PIK3C2A,GRB2,CREBBP,MDM2,NFKB2,CREB3L4,SIN3A,RRAS2,PTPN11,IRS1,CDKN1A,E2F1,CDKN1B
Glioblastoma Multiforme Signaling	0.00057544	3.24E+00	PIK3CA,RALA,AXIN1,NF2,FZD1,PDGFC,E2F6,AKT1,RHOB,RHOD,PIK3C3,PIK3CG,TSC2,AKT3,FZD2,WNT5B,EGFR,AKT2,PIK3C2A,GRB2,ITPR2,MDM2,RHOJ,ITPR1,APC,RHOQ,RRAS2,PTPN11,NF1,RND3,IRS1,E2F1,CDKN1A,FZD6,PLCB3,FZD5,CDKN1B,FZD7
Production of Nitric Oxide and Reactive Oxygen Species in Macrophages	0.000645654	3.19E+00	APOE,PIK3CA,MAP3K11,PPP2R5B,MAP3K5,MAPK13,NFKB1,AKT1,PPP1R10,RHOB,RHOD,PIK3CG,PIK3C3,AKT3,PPP2R2C,NFKBIB,PRKCA,AKT2,MAP2K7,PIK3C2A,GRB2,TYK2,CREBBP,IFNGR1,

			PPP1R11,RHOJ,PCYOX1,NFKB2,TLR2,MAP3K12,PPP2R1A,MAPK14,RHOQ,PTPN11,RND3,PTPA,PPP2R3A,IRS1,CAT,PPP2R5E,PPP2R1B,SIRPA
Ceramide Signaling	0.000691831	3.16E+00	AKT2,PIK3CA,RALA,BAD,PIK3C2A,GRB2,PPP2R5B,SPHK2,SMPD1,NFKB2,NFKB1,DIABLO,CTSD,PPP2R1A,AKT1,RRAS2,PTPN11,PTPA,PPP2R3A,IRS1,PIK3C3,PIK3CG,AKT3,PPP2R2C,PPP2R5E,PPP2R1B
p53 Signaling	0.000707946	3.15E+00	PIK3CA,HIF1A,ST13,EP300,CCNG1,CASP6,AKT1,STAG1,PIK3CG,PIK3C3,PPP1R13B,CCNK,AKT3,AKT2,PIK3C2A,TOPBP1,GRB2,MED1,CSNK1D,PERP,MDM2,SERPINE2,MAPK14,PTPN11,IRS1,CDKN1A,E2F1,PML
Non-Small Cell Lung Cancer Signaling	0.000707946	3.15E+00	PIK3CA,AKT2,RALA,BAD,PIK3C2A,GRB2,ITPR2,PDPK1,ITPR1,SIN3A,AKT1,RRAS2,PTPN11,IRS1,PIK3CG,PIK3C3,E2F1,TGFA,AKT3,ERBB2,RXRB,EGFR,PRKCA
Colorectal Cancer Metastasis Signaling	0.000707946	3.15E+00	IL6ST,PRKACB,PIK3CA,RALA,GRK2,BAD,AXIN1,MSH3,MMP14,SMAD3,LRP6,FZD1,NFKB1,PDGFC,PTGER1,VEGFA,AKT1,RHOB,RHOD,PIK3C3,PIK3CG,AKT3,FZD2,WNT5B,EGFR,SMAD2,LRP5,AKT2,PIK3C2A,GRB2,GNG2,TYK2,IFNGR1,RHOJ,NFKB2,APC,TLR2,CDH1,RRAS2,PRKAR2B,RHOQ,MSH2,PTPN11,RND3,IRS1,FZD6,PRKACA,FZD5,ADCY7,TCF7L2,FZD7
IGF-1 Signaling	0.000794328	3.10E+00	PRKACB,SOCS3,IGFBP4,PIK3CA,RALA,BAD,SOCS6,PDPK1,YWHAQ,AKT1,PIK3CG,PIK3C3,AKT3,SOCS2,CSNK2B,PXN,AKT2,PIK3C2A,YWHAE,GRB2,PRKAR2B,RRAS2,NOV,PTPN11,IRS1,IGFBP3,PRKACA,SOCS5
Huntington's Disease Signaling	0.000870964	3.06E+00	Hspa1b,HSPA1A/HSPA1B,PDPK1,HDAC10,CTSD,PIK3CG,NAPA,DNM2,POLR2I,AKT2,HDAC4,GNG2,CREBBP,CREB3L4,ITPR1,HDAC5,DNAJC5,PTPN11,CAPN1,CASP2,IRS1,HTT,PSME4,PLCB3,PIK3CA,GNA14,VTI1B,EP300,CASP6,ARFIP2,AKT1,SP1,PIK3C3,AKT3,DNAJB1,CASP8,EGFR,PRKCA,MAP2K7,YKT6,PIK3C2A,GRB2,GNAQ,HSPA2,SIN3A,POLR2G,PSME1,CAPNS1,HAP1,NCOR2
Paxillin Signaling	0.000912011	3.04E+00	PIK3CA,RALA,ARF1,CRK,NCK1,MAPK13,BCAR1,NCK2,ITGA3,ARF6,ARFIP2,PIK3C3,PIK3CG,ITGAV,VCL,ACTA1,ACTN1,PAK4,PARVA,PXN,PIK3C2A,GRB2,GIT2,RRAS2,MAPK14,PTPN11,IRS1,ITGA1,ACTN4

Pathway analyses of genes with allelic ratio outliers reveal enrichment in genes that define the cancerous state. Genes (2634) marked by eSNVs with ratio outliers in one or more tumors (see Supplementary Data) were analyzed in IPA software (Qiagen). Listed are the canonical pathways (P < 0.001) when corrected for multiple hypothesis testing based on the Benjamini-Hochberg (B-H) method available in the software package[2].

SUPPLEMENTARY TABLE 2

Ingenuity Canonical Pathway	B-H p-value	Molecules
Antigen Presentation Pathway	2.62 E-04	B2M, CALR, CD74, HLA-A, HLA-DMA, HLA-DQA1, HLA-DQB1, HLA-DRB5, PSMB9, TAP1, TAP2, TAPBP
Endocannabinoid Cancer Inhibition Pathway	1.28 E-02	ADCY7, AKT1, AKT1S1, CASP6, CASP8, CDH1, EIF2A, GNA13, HIF1A, MAP2K7, MAPK14, PDGFC, PIK3C3, PIK3CA, PIK3CG, PRKAB1, PRKAR2B, PTPN11, ROCK2, SMPD1, SPTLC2, TCF7L2
Reelin Signaling in Neurons	2.21 E-02	AKT1, APP, ARHGEF2, ARHGEF5, ARHGEF11, ARHGEF12, ITGA3 MAP2K7, MAPK8IP3, PAFAH1B1, PIK3C3, PIK3CA, PIK3CG, PTPN11, VLDLR
mTOR Signaling	2.21 E-02	AKT1, AKT1S1, ARHGAP8, EIF3E, EIF3G, EIF4A3, FAU, HIF1A, MAPKAP1, PDGFC, PDPK1, PIK3C3, PIK3CA, PIK3CG, PLD4, PPP2R1A, PPP2R1B, PRKAB1, PRR5, PTPN11, RHOQ, RPS18, RPS27L, RPS6KA4, TSC2
Molecular Mechanisms of Cancer	2.95 E-02	ADCY7, AKT1, APC, ARHGEF2, ARHGEF5, ARHGEF11, ARHGEF12, ARHGEF18, AURKA, AXIN1, BID, BMPR2, CASP6, CASP8, CDC25A, CDH1, CDK10, CTNND1, FZD2, FZD5, GNA13, HAT1, HIF1A, ITGA3, LRP6, MAPK14, MDM2, NF1, NFKB2, NFKBIB, PIK3C3, PIK3CA, PIK3CG, PRKAR2B, PTPN11, RHOQ, SMAD5 SUFU, TYK2

Canonical Pathways from IPA analysis of genes with eSNV outliers at a Z-score threshold = 2.5. Genes with allelic ratio outliers defined by increasing Z-score threshold to 2.5 still found in pathways associated with cancer. Genes (1291) from Supplementary Table 1 which continued to meet the criteria of containing eSNV outliers when the threshold Z-score was increased from 2 to 2.5 (~ P = 0.0124) were analyzed in IPA. Listed are the top canonical pathways in the report, the genes falling into those pathways, and the corresponding p-values corrected for multiple hypothesis testing.

Supplementary References

1. Felts, S. J. *et al.* Widespread non-canonical epigenetic modifications in MMTV-NeuT breast cancer. *Neoplasia* **17**, 348-357, doi:10.1016/j.neo.2015.02.006 (2015).

2. Benjamini, Y. & Hochberg, Y. Controlling the false discovery rate: a practical and powerful approach to multiple testing. *J. Roy. Statist. Soc. Ser.* **B 57**, 289-300 (1995).
