# Supplementary material for: Stochastic changes in gene expression promote chaotic dysregulation of homeostasis in clonal breast tumors
Source: Commun Biol. 2019 Jun 14;2:206. doi: 10.1038/s42003-019-0460-0 (PMC6570763; doi:10.1038/s42003-019-0460-0)
Supplement: Supplementary file 2 — Description of Additional Supplementary Items [file 42003_2019_460_MOESM2_ESM.docx]

**Supplementary Data 1.** Genes marked by eSNVs with ratio outliers in one or more tumors were tabulated and characterized according to Gene Symbol, Entrez Gene Name, and the subcellular localization and function (where known) of their encoded products using IPA analysis software. Genes in bold match those reported recently as breast cancer susceptibility genes as referenced in the text.

**Supplementary Data 2.** Correlation results testing genes expressed in F1 tumors as a function of the number of allelic outliers. Using data from the 12 F1 breast tumors that lacked evidence of linked eSNV being skewed toward one parent, the number of eSNV outliers in each tumor was quantitated and tested for correlation with gene expression levels in those tumors (using all genes expressed at least 0.5 RPKM log_2_). The results from this ANOVA (Partek Genomics Suite 6.6) are tabulated based on highest correlation coefficient (r).

**Supplementary Data 3.** Mean BALB/c-allelic frequencies (CAF) were calculated for eSNV where the minor allele raw count was greater than 15 and shown in Supplemental Figure 1 for each chromosome. For added reference, the chromosome location, Ensembl ID, and Gene symbol of each eSNV are included.

**Supplementary Data 4.**  For Figure 1 (top), mean CAF for the original parental counts were calculated for 308 eSNV on ChrX on a tumor-by-tumor basis and illustrated using Prism. Mean CAF < 0.25 or > 0.75 are indicated as clonal in the figure.

**Supplementary Data 5.**  For Figure 1 (bottom), mean CAF for the original parental counts were calculated for 446 eSNV on Chr9 on a tumor-by-tumor basis and illustrated using Prism.

**Supplementary Data 6.** Spectral Karyotype (SKY) analysis was performed by the Mayo Clinic Cytogenetics Core using primary cultures of F1 mouse breast tumors. Quantitation of chromosomal aberrations is summarized in Table 1 for 100 mitotic figures examined (10 per individual culture).

**Supplementary Data 7.** From the CAF of over 11,000 eSNV for each tumor sample, Z-scores were calculated in Excel (Z = (individual CAF minus mean CAF (N=20) (StdDev)^-1). In Supplementary Figure 3, individual data cells containing calculated Z-scores were shaded as white (Z-score > 2), black (Z-score < -2), or otherwise gray. eSNV with one outlier were considered further as described in the text.

**Supplementary Data 8.** A subset of data from Supplementary Figure 3 were used to make Figure 3 a)-c) in order to illustrate how CAF and Z-score are related and how allelic ration outliers were defined.

**Supplementary Data 9.**  Gene expression data in RPKM log2 as a function of the number of eSNV in V-LOH for 12 tumors is shown in Figure 4a for the top 30 genes. The results of the correlation analysis are reported in Supplementary Data 2.

**Supplementary Data 10.** Shown is the full report of correlation (r) values between the number of eSNV in V-LOH and gene expression which were then placed (binned) into 0.1 intervals using Prism and shown in Figure 4b.

**Supplementary Data 11.**  Shown in Figure 4c are the gene expression data in RPKM log2 for the top 30 genes and the number of eSNV in V-LOH for 12 tumors tested in correlation analysis when the tumor order in the analysis was randomized.
