# Supplementary material for: Stochastic changes in gene expression promote chaotic dysregulation of homeostasis in clonal breast tumors
Source: Commun Biol. 2019 Jun 14;2:206. doi: 10.1038/s42003-019-0460-0 (PMC6570763; doi:10.1038/s42003-019-0460-0)
Supplement: Supplementary file 3 — Reporting Summary [file 42003_2019_460_MOESM3_ESM.pdf]

nature research

Corresponding author(s): Larry R. Pease, Ph.D.

Last updated by author(s): 04/05/2019

Reporting Summary

Nature Research wishes to improve the reproducibility of the work that we publish. This form provides structure for consistency and transparency in reporting. For further information on Nature Research policies, see Authors & Referees and the Editorial Policy Checklist.

Statistics

For all statistical analyses, confirm that the following items are present in the figure legend, table legend, main text, or Methods section.

n/a	Confirmed	
☐	☒	The exact sample size (*n*) for each experimental group/condition, given as a discrete number and unit of measurement
☒	☐	A statement on whether measurements were taken from distinct samples or whether the same sample was measured repeatedly
☐	☒	The statistical test(s) used AND whether they are one- or two-sided<br>*Only common tests should be described solely by name; describe more complex techniques in the Methods section.*
☒	☐	A description of all covariates tested
☐	☒	A description of any assumptions or corrections, such as tests of normality and adjustment for multiple comparisons
☐	☒	A full description of the statistical parameters including central tendency (e.g. means) or other basic estimates (e.g. regression coefficient) AND variation (e.g. standard deviation) or associated estimates of uncertainty (e.g. confidence intervals)
☐	☒	For null hypothesis testing, the test statistic (e.g. *F*, *t*, *r*) with confidence intervals, effect sizes, degrees of freedom and *P* value noted<br>*Give P values as exact values whenever suitable.*
☒	☐	For Bayesian analysis, information on the choice of priors and Markov chain Monte Carlo settings
☒	☐	For hierarchical and complex designs, identification of the appropriate level for tests and full reporting of outcomes
☐	☒	Estimates of effect sizes (e.g. Cohen's *d*, Pearson's *r*), indicating how they were calculated

Our web collection on statistics for biologists contains articles on many of the points above.

Software and code

Policy information about availability of computer code

Data collection	All data were collected using public or commercially available software: MapRseq 2.0.0; TopHat v2; GenASis software v7.27 and CytoVision Imaging software version 7.4 (Leica)
Data analysis	All data analysis used public or commercially available tools: Excel for Microsoft Office 2010; Partek Genomics Suite 6.6; Prism 7 (GraphPad); Ingenuity Pathway Analysis (licensed by Qiagen) and Gene Ontology (public online consortium)

For manuscripts utilizing custom algorithms or software that are central to the research but not yet described in published literature, software must be made available to editors/reviewers. We strongly encourage code deposition in a community repository (e.g. GitHub). See the Nature Research guidelines for submitting code & software for further information.

Data

Policy information about availability of data

All manuscripts must include a data availability statement. This statement should provide the following information, where applicable:

- Accession codes, unique identifiers, or web links for publicly available datasets
- A list of figures that have associated raw data
- A description of any restrictions on data availability

The data for this study have been deposited in the NCBI Gene Expression Omnibus (GEO) database under the GEO accession number GSE128775.

Field-specific reporting

Please select the one below that is the best fit for your research. If you are not sure, read the appropriate sections before making your selection.

☒ Life sciences ☐ Behavioural & social sciences ☐ Ecological, evolutionary & environmental sciences

Life sciences study design

All studies must disclose on these points even when the disclosure is negative.

Sample size	Sample number (animals and tumors per animal) were chosen based on the assumption that random ability to measure outliers (the topic of this study) is 1 in 20. In addition, for optimal genome-wide coverage of gene expression with available resources, RNA sample number analyzed per lane was chosen to maximize RNAseq read depth and read quality.
Data exclusions	RNAseq reads were trimmed from 150 bases to 100 bases to maximize read quality. No data was excluded from the manuscript. Certain analyses focused on data subsets as clearly explained in the manuscript.
Replication	No attempt was made to replicate this study, as the dataset contains thousands of measures. The overall findings of this study replicate previous findings in which a completely different sample type and different type of data were analyzed.
Randomization	In some analyses, a random sample of the original data was used to test the significance of our findings. These random selections used random number generator available in Excel.
Blinding	The samples used (whether animals, tumor from those animals, or RNA from those tumors) formed a single group and were identical in appearance and thus analyzed as a set. There was no blinding.

Reporting for specific materials, systems and methods

We require information from authors about some types of materials, experimental systems and methods used in many studies. Here, indicate whether each material, system or method listed is relevant to your study. If you are not sure if a list item applies to your research, read the appropriate section before selecting a response.

Materials & experimental systems

n/a	Involved in the study	
☒	☐	Antibodies
☒	☐	Eukaryotic cell lines
☒	☐	Palaeontology
☐	☒	Animals and other organisms
☒	☐	Human research participants
☒	☐	Clinical data

Methods

n/a	Involved in the study	
☒	☐	ChIP-seq
☒	☐	Flow cytometry
☒	☐	MRI-based neuroimaging

Animals and other organisms

Policy information about studies involving animals; ARRIVE guidelines recommended for reporting animal research

Laboratory animals	Laboratory mice strains BALB/c-neuT and FVB/J were inter-crossed to generate F1 progeny. Females up to 20 weeks of age were used in this breast cancer study.
Wild animals	This study did not use wild animals.
Field-collected samples	This study did not collect samples from the field.
Ethics oversight	Studies are IACUC evaluated and approved.

Note that full information on the approval of the study protocol must also be provided in the manuscript.
